# Editorial: Dysregulation of Th17 and Treg cells in autoimmune diseases

**DOI:** 10.3389/fimmu.2023.1151836

**Published:** 2023-02-14

**Authors:** Anne L. Astier, David M. Kofler

**Affiliations:** ^1^ Toulouse Institute for Infectious and Inflammatory Diseases (Infinity), National Institute of Health and Medical Research (INSERM) U1291, CNRS U5051, Université de Toulouse III, Toulouse, France; ^2^ Division of Rheumatology and Clinical Immunology, Department I of Internal Medicine, Faculty of Medicine and University Hospital Cologne, University of Cologne, Cologne, Germany; ^3^ Center for Integrated Oncology Aachen Bonn Cologne Duesseldorf, Cologne, Germany

**Keywords:** regulatory T cells, Th17 cells, autoimmune diseases, immune homeostasis, type 1 diabetes, rheumatoid arthritis, psoriasis vulgaris, multiple sclerosis

Immune homeostasis is key to maintain a healthy state and this results from a balance between effector and regulatory T (Treg) cells (1). In particular, Th17 cells and Treg cells have opposing functions and these two subsets play a key role in the maintenance of immune homeostasis. Their stability, plasticity and function are dysregulated in a large array of autoimmune diseases, in which an imbalance between pathogenic Th17 and Treg cells was observed. However, the molecular mechanisms responsible for Th17 and Treg cell dysregulation remain largely unknown.

The development of novel treatments aiming at restoring the balance between these subsets is therefore being investigated. However, one of the key issues is the plasticity of these T helper subsets, and maintenance of their phenotype will be a major aspect to be addressed. In this Research Topic, we aimed to address Treg/Th17 dysregulation by bringing together some of the latest developments in the field. A better characterization of the mechanisms involved in the stability of T cell subsets may allow the identification of new targets for the treatment of autoimmune diseases.

Herein, several researchers report new findings on Th17 cells and Treg cells and describe their role in the pathogenesis of autoimmune diseases such as type 1 diabetes, psoriasis vulgaris, multiple sclerosis, and systemic lupus erythematosus. One of the first challenges is a better characterization of distinct T helper cell subsets. The manuscript by Corte-Real et al. discusses CSF2RB/CD131 as a potential additional marker for Tregs and its increased expression in patients with multiple sclerosis and systemic lupus erythematosus. Treg therapies could provide exciting means to revert autoimmunity (2, 3). One of the challenges is the lack of antigen specificity of Treg cells after polyclonal expansion. The challenges and expectations of generating CAR-Tregs is discussed by Riet and Chmielewski. In their articles Starosz et al. and Kim et al. focus on the imbalance between Treg cells and Th17 cells in autoimmune diseases. Starosz et al. found a significantly higher Treg/Th17 ratio in pediatric patients with newly diagnosed type 1 diabetes as compared to healthy individuals. Interestingly, lower HbA1c and daily insulin requirement are associated with higher Treg cell levels. Moreover, Th17 cell levels correlate with c-peptide levels after two years of disease. Kim et al. review the current knowledge about Th17 cells and Treg cells in psoriasis vulgaris and highlight Th17 cell plasticity as well as differences between IL-17A and IL-17F. Hu et al. report differences between the transcriptome of pathogenic Th17 cells and non-pathogenic Th17 cells. Single-cell transcriptomic profiling has revealed molecular heterogeneity within immune cell subsets and has revolutionized systems immunology (4). Hu et al. revealed that the transcriptome of Th17 cells from individuals with autoimmune diseases contains different regions of accessible chromatin. The results presented by Hu et al. show how pathogenicity of Th17 cells can be regulated at the chromatin level. Bednar et al. focus on Treg cells in autoimmunity and present insights into intrinsic brake mechanisms driving pathogenesis and immune homeostasis. These mechanisms include FOXP3 stability, CTLA-4 and cytokines in the inflamed tissue.

Altogether, the articles in this Research Topic provide a comprehensive update on the current knowledge about Treg cell stability, gene therapy for autoimmune diseases involving Treg cells and the impaired Treg/Th17 cell balance in autoimmune diseases. Moreover, previously unknown characteristics of Treg cells and Th17 cells are described and the role of both subsets for the development of autoimmune diseases is highlighted. We would also like to thank all the contributors to this topic and the reviewers for the time they spent to make this Research Topic possible.

## Author contributions

All authors listed have made a substantial, direct, and intellectual contribution to the work and approved it for publication.
